# Does the eight-factor “power to live” in disaster exist since childhood?

**DOI:** 10.3389/fpubh.2022.1022939

**Published:** 2022-12-12

**Authors:** Yutaka Matsuzaki, Ryo Ishibashi, Mari Yasuda, Azumi Tanabe-Ishibashi, Akio Honda, Tsuneyuki Abe, Motoaki Sugiura

**Affiliations:** ^1^Institute of Development, Aging, and Cancer, Tohoku University, Sendai, Japan; ^2^Smart-Aging Research Center, Tohoku University, Sendai, Japan; ^3^International Research Institute of Disaster Science, Tohoku University, Sendai, Japan; ^4^Department of Information Design, Faculty of Informatics, Shizuoka Institute of Science and Technology, Fukuroi, Japan; ^5^Graduate School of Arts and Letters, Tohoku University, Sendai, Japan

**Keywords:** children, personality, disaster prevention education, emotion regulation, leadership, problem-solving, factor structure

## Abstract

**Background:**

Studies on the survivors of the 2011 Great East Japan earthquake and tsunami have revealed eight factors, called power to live, which are closely related to resilience and effective coping after intense and prolonged stress. However, whether the eight factors, which were examined in adults, are applicable to children is unclear. The purpose of this study is to evaluate whether the eight-factor structure of power to live was present since late childhood.

**Method:**

A 34-item power to live questionnaire was filled by middle- to upper-grade elementary (*n* = 378) and junior high school students (*n* = 456). Moreover, because elementary school students may lack introspective ability, their power to live was evaluated through a parental assessment (*n* = 358). Additionally, we examined the relationship between each power to live factor and questions regarding disaster prevention awareness among 25 elementary school students.

**Results:**

The results from confirmatory factor analysis for factor structure revealed generally acceptable fit indices. The reports from elementary school students and their parents significantly positively correlated with each power to live factor. Although reliability indices for factors such as stubbornness, etiquette, self-transcendence, and active well-being were not good for elementary school students, the reliability indices for all factors, excluding stubbornness, increased in junior high school students. Moreover, we identified a correlation between problem-solving, altruism, and emotional regulation and questionnaire items regarding awareness of disaster prevention in elementary school students.

**Conclusion:**

Our results suggest that although factors common to adults, such as leadership, problem-solving, altruism, and emotional regulation, were identified at the elementary school stage, some factors, such as stubbornness, are in the process of being formed. Future studies should examine the developmental changes assumed to underlie these factors and their relationship to experience and neurodevelopmental basis.

## Introduction

Resilience is defined as the capacity of a dynamic system to withstand or recover from significant challenges that threaten its stability, viability, and development (1, 2). Resilience from a catastrophic disaster involves the diverse and long-term process of evacuation, shelter-living, and rebuilding lives. Examining the factors that play a protective role in these processes is necessary when considering disaster prevention and promoting resilience (3).

From the studies on survivors of the 2011 Great East Japan earthquake and tsunami, which killed nearly 19,000 people and caused extensive damage to the coastal areas of eastern Japan, eight factors called “power to live” were extracted that are closely related to resilience and effective coping using a bottom-up approach (4, 5). Sato et al. (4) interviewed survivors of the 2011 Great East Japan earthquake and tsunami about their experiences of avoiding crises and solving problems in various disaster contexts, such as evacuation, living in shelters, and rebuilding lives, as well as the psychological and behavioral characteristics that worked to their advantage. Based on that survey, Sugiura et al. (5) conducted a questionnaire survey of 1,412 disaster survivors and constructed an 8-factor, 34-item power to live questionnaire using exploratory factor analysis and examined the relationship between each factor and behavior during the 2011 Great East Japan earthquake and tsunami.

The power to live questionnaire is an eight-factor questionnaire that evaluates a wide-range of individual characteristics related to post-disaster resilience. The eight factors include: Factor 1 (leadership) represents the attitude and habit of gathering and organizing people; Factor 2 (problem-solving) is associated attitudes and practices that strategically address problems; Factor 3 (altruism) is a personality trait that leads to concern for and assistance of others; Factor 4 (stubbornness) is the attitude or habit of sticking to one's wishes or beliefs; Factor 5 (etiquette) is the attitude or habit of following social conventions in one's daily behavior; Factor 6 (emotional regulation) is the attitude or habit of attempting to calm down in a difficult environment; Factor 7 (self-transcendence) is associated with consciousness and making sense of life from a spiritual perspective; and Factor 8 (active well-being) is related to the daily practice of maintaining and improving physical, mental, and intellectual health (5).

Each factor in the power to live questionnaire was examined in relation to the survivors' condition at the time of the 2011 Great East Japan earthquake and afterwards. Promptness during evacuation positively correlated with leadership, problem-solving, and emotional regulation (5, 6). Positive correlations have been observed in other indicators such as problem-solving in shelters (problem-solving, altruism, emotional regulation, and self-transcendence) or subjective sense of recovery (problem-solving and active wellbeing) (5). Leadership, emotional regulation, problem-solving, etiquette, and self-transcendence correlated with rebuilding of houses (7). Relationships were also found between post-disaster problem-solving styles (e. g., self-resolution, resolution through request, resolution through acquaintances, etc.) and various factors in the power to live questionnaire (8).

Studies have evaluated the neurocognitive basis of each factor in the power to live questionnaire (9, 10). In a study in which an MRI task was used to simulate easy or difficult problems in the operation of powerplants, a relationship was found between brain activity involved in coping with difficult problems and problem-solving (11). In another study, an inverse correlation was reported between neural activation during constructive thinking and stubbornness (12).

However, whether the eight factors related to the power to live examined in adult survivors from the 2011 Great East Japan earthquake and tsunami are applicable to children is unclear. In disaster prevention education, knowledge acquisition is often the main goal, and focusing on nurturing the characteristics required to overcome a disaster is challenging (13–15). Evaluating the similarities and differences between power to live factors in adults and children can help identify traits formed at an early age, which can then be applied to disaster-prevention education. Although protective factors, such as cognitive skills (16, 17) and family relationships (18) have been related to resilience in children, children differ from adults in many traits related to resilience, including nurturing of family, community factors, and intrapersonal factors, such as immature personality and cognitive functioning (19). There is a possibility of prematurity in each power to live factor between elementary and junior high school students.

Thus, the purpose of this study is to confirm whether the eight-factor structure of the power to live, which was reported for the adult survivors of the 2011 Great East Japan earthquake and tsunami, is present since late childhood. For this purpose, we examined the factor structure and homogeneity of the power to live in elementary and junior high school students. Because the questionnaire may be unsuitable for students, especially for elementary school students given their ability of introspection, elementary school students were assessed by using a combination of the self- and parent-rated power to live questionnaires. Moreover, the items were paraphrased to match the vocabulary of elementary school students to not detract from the original meaning. In addition to the original 34-item version, the model was examined using a 16-item version created by extracting two items that showed higher correlation with each factor score (20). Scores on the 16-item version were shown to have a very high positive correlation with the 34-item version, and its relevance to behavior during the 2011 Great East Japan earthquake and tsunami was also examined (20). Moreover, we explored the relationship between disaster prevention awareness and the power to live among elementary school students to apply them on disaster prevention education.

## Methods

### Participants

In the Tohoku region of Japan, envelopes containing questionnaires were distributed and collected on a voluntary basis from elementary and junior high schools. The envelopes contained a written explanation of the questionnaire and a statement that the return of the envelope constituted consent. The elementary schools distributed questionnaires to families with children in grades 3 and above. The questionnaires were collected from 455 families at the elementary school. Of these, 378 (191 boys and 187 girls, 9–12 years old, mean age = 10.71 years, standard deviation = 0.84), without missing responses from the children, were included in the data analysis for the elementary school students themselves. In addition, data from 358 parent–child pairs, who also had no missing responses, were used for analyzing the relationship between their own ratings and those of their parents. Questionnaires filled out by the junior high school students themselves were distributed at 4 schools and collected from 513 students. The 456 students whose answers were not missing were included in data analysis (237 boys and 219 girls, 12–16 years old, mean age = 13.58 years, standard deviation = 0.99). The participants of the questionnaire-based survey on disaster prevention awareness were obtained from 28 sixth graders, 25 of whom had no missing responses on both power to live questionnaires, and the relationship between the two scales was examined (9 boys and 16 girls, aged 11–12 years; mean age = 11.92 years, standard deviation = 0.28). The procedures for this study were approved by the Institutional Review Board of the International Research Institute of Disaster Science, Tohoku University, Japan.

### Questionnaires

#### Power to live questionnaire

The power to live questionnaire (5) was used for the survey. The questionnaire contained 34 items (Table 1), asking for responses on a 6-point scale ranging from “0 = not at all” to “5 = very much.” From the responses, individual scores are calculated for eight factors related to the power to live. As noted earlier, the eight factors were leadership, problem solving, altruism, stubbornness, etiquette, emotional regulation, self-transcendence, and active well-being. Only each factor was considered, and no higher-order factors were assumed. Cronbach's alpha for each factor ranges from 0.71 to 0.80.

**Table 1:** Power to live questionnaire and factor loading in elementary and junior high school students (34-item factor structure).

**Item**	**Elementary school student**	**Junior high school student**
**Factor 1. Leadership**		
1. To resolve problems, I gather together everyone involve to discuss the matter	0.71	0.70
2. In everyday life, I often take the initiative to gather people together	0.79	0.82
3. I take the initiative in taking to other people	0.62	0.79
4. Sophisticated words that move people's hearts come out of my mouth	0.70	0.55
5. In everyday life, I make sure to keep in contact with friends and acquaintances	0.52	0.81
**Factor 2. Problem-solving**		
6. When I am fretting about what I should do, I compare several alternative actions	0.66	0.59
7. Before taking action, I think of a plan and the order of priority	0.69	0.83
8. When talking to someone, I think about that person's personality, wishes, and abilities and choose an appropriate attitude and words accordingly	0.58	0.66
9. The more agitated the people around me become, the calmer I somehow become	0.32	0.71
10. To resolve a problem, I first of all initiate action	0.65	0.63
**Factor 3. Altruism**		
11. I like it when other people rely on me and are grateful to me	0.82	0.79
12. When I see someone having trouble, I have to help them	0.65	0.52
13. When someone asks me to do something for them, I cannot refuse	0.35	0.85
14. Other people's good fortune makes me happy so I like to help others	0.87	0.67
15. I am meddlesome and I like to do things for others	0.61	0.45
**Factor 4. Stubbornness**		
16. I am stubborn and always get my own way	0.54	0.56
17. I unhesitatingly say whatever it is I want to say	0.50	0.69
18. I clearly distinguish between black and white: what's good is good, and what's bad is bad	0.50	0.50
19. I hate losing	0.65	0.54
20. I am highly motivated with regard to things that I like or want to d.	0.59	0.44
**Factor 5. Etiquette**		
21. On a daily basis, I take the initiative in greeting family members and people living in the neighborhood	0.67	0.71
22. In everyday life, I take care of myself as much as possible	0.59	0.70
23. When someone has helped me or been kind to me, I clearly convey my feelings of gratitude	0.63	0.61
**Factor 6. Emotional regulation**		
24. During difficult times, I endeavor not to brood	0.71	0.74
25. During difficult times, I endeavor to think positively, telling myself that this experience will benefit me in the future	0.72	0.67
26. During difficult times, I compare my circumstances with the situation around me and in society, and I think that matters cannot be helped	0.58	0.39
27. When something happens, I try to stay calm and not panic	0.71	0.76
**Factor 7. Self-transcendence**		
28. I am aware that I am alive, and have a sense of responsibility in living	0.56	0.80
29. I am aware of the path and teachings I should follow as a person	0.61	0.64
30. I am aware of the role I should play in society	0.60	0.62
31. I think that my actions toward others will go around and eventually come back to me	0.56	0.77
**Factor 8. Active well-being**		
32. In everyday life, I have habitual practices that are essential for relieving stress of giving me a change of pace	0.53	0.60
33. In everyday life, I have habitual practices that are essential for maintaining my physical health	0.55	0.78
34. In everyday life, I endeavor to find opportunities to acquire new knowledge, skills, and attitudes	0.61	0.76

Original wording was used for junior high school students. The questionnaire items for the elementary school students were rephrased to fit their vocabulary and comprehension, and all items were drafted by all authors based on the 34-item version of the questionnaire. Then, we interviewed several third-grade elementary school students—the lower limit of the surveyed grade level—to evaluate their understanding of the words used in the items and determined the wording of the final questionnaire. Original wording was used for the parent of the elementary school student, with instructions to answer and respond to the question “How much does this apply to your child?”

#### Disaster prevention awareness

Questionnaire items were selected from those used in studies on disaster prevention education (21–23). Table 2 shows the eight questions asked of the children. The children were asked to self-evaluate their evacuation behavior and sense of fear as relevant factors, as well as their proactive involvement in disaster prevention and self-evaluation of communication with family members involved in disaster prevention. Each question was answered on a 5-point scale ranging from “1 = Strongly Disagree” to “5 = Strongly Agree.”

**Table 2:** Correlation coefficients between disaster prevention awareness and power to live factors (34-item version).

**Item**	**Leader-ship**	**Problem-solving**	**Altruism**	**Stubborn-ness**	**Etiquette**	**Emotional regulation**	**Self-Transcen-dence**	**Active well-being**
Q1. Do you think you could evacuate safely if an earthquake, tsunami, typhoons, heavy rain, or volcanic eruption were to occur?	0.30	0.22	0.48^*^	−0.14	0.32	0.29	0.32	−0.13
Q2. Do you think your family could evacuate safely if an earthquake, tsunami, typhoons, heavy rain, or volcanic eruption were to occur?	−0.04	0.10	0.41^*^	−0.14	0.00	−0.04	0.26	−0.11
Q3. Are you scared of natural disasters, like earthquakes, tsunamis, typhoons, heavy rain, and volcanic eruptions?	−0.15	−0.10	−0.27	−0.07	−0.11	−0.42^*^	−0.21	−0.30
Q4. Do you think you may get injured if a natural disaster were to occur?	0.04	0.03	0.10	−0.20	0.27	−0.09	0.08	0.03
Q5. Do you think natural disasters will occur in your area?	0.30	0.46^*^	0.31	−0.25	0.41^*^	0.33	0.23	0.26
Q6. Do you think you need to talk with your family to decide what to do in the event of a natural disaster?	0.09	−0.11	0.17	0.22	0.11	−0.01	0.41^*^	0.13
Q7. Do you think your family would become safer if you talked and made promises to improve preparedness?	0.17	0.26	0.26	−0.18	0.47^*^	0.03	0.45^*^	0.05

* p < 0.05.

### Statistical analyses

Confirmatory factor analysis (CFA) was conducted on three data sets (elementary school students, parents of elementary school students, and junior high school students). CFA verifies the validity of the factor structure from the goodness-of-fit indices of the model. Two models were examined: a 34-item version (5) and a 16-item version (20). The maximum likelihood method was used to estimate factor loadings. This study reports on the comparative fit index (CFI), Tucker–Lewis index (TLI), root-mean-square error of approximation (RMSEA), and standardized root-mean-square residual (SRMR). In a study, goodness-of-fit was considered good if CFI > 0.95, TLI > 0.95, RMSEA < 0.06, and SRMR < 0.06 (24).

The similarity of the factor structure between elementary and junior high school students was tested by employing multigroup CFA. Goodness-of-fit indices were calculated and compared across models for factor structure (configural model), factor loading (weak invariance model), and when factor loading and intercept (strong configural) were constrained to be equal. Additionally, correlational analyses (Pearson's *r*) were conducted to explore the relationship between each factor on the power to live questionnaire and responses to questions on disaster prevention awareness. Software (ver. 4. 2. 1) was used for statistical analysis. Confirmatory factor analysis was performed using the lavaan package (ver. 0. 6–12).

## Results

### Descriptive statistics, reliability in the 34-item version power to live questionnaire

The means and standard deviations for each factor of the 34-item version of the power to live questionnaire and values of Cronbach's alpha are shown in Table 3. Cronbach's alpha for each factor in elementary school students ranged from 0.58 to 0.80. Reliability coefficients were slightly lower for stubbornness (0.68), etiquette (0.66), self-transcendence (0.67), and active wellbeing (0.58). By contrast, reliability coefficients were acceptable (≥0.70) for leadership, problem-solving, altruism, and emotional regulation. For junior high school students, although slightly below the customary criterion for stubbornness (0.67), the other factors of power to live showed acceptable reliability (from 0.72 to 0.85).

**Table 3:** Descriptive statics and Cronbach's alpha of the power to live questionnaire (34-item factor structure).

		**Elementary school students**			**Junior high school students**	
	***M***	**SD**	**α**	***M***	**SD**	**α**
1. Leadership	14.22	4.94	0.80	15.86	5.63	0.85
2. Problem-solving	14.22	4.57	0.70	17.64	4.66	0.82
3. Altruism	15.85	4.98	0.79	16.82	4.53	0.79
4. Stubbornness	15.30	4.55	0.68	15.88	4.12	0.67
5. Etiquette	10.96	2.82	0.66	12.44	2.46	0.72
6. Emotional regulation	10.70	3.84	0.76	13.55	3.83	0.73
7. Self-transcendence	13.66	3.57	0.67	13.64	4.10	0.81
8. Active well-being	8.97	3.26	0.58	10.20	3.25	0.76

### Confirmatory factor analysis and factor loading in the 34-item version of the power to live questionnaire

CFA results are summarized in Table 4. For elementary school students, the goodness of fit indices were CFI = 0.84, TLI = 0.82, RMSEA = 0.06, and SRMR = 0.07. The trend in the goodness-of-fit index was the same for junior high school students (CFI = 0.88, TLI = 0.87, RMSEA = 0.06, and SRMR = 0.07). Regarding standardized factor loading on each item, junior high school students generally exceeded the conventional criterion (>0.40); however, only item 26 was slightly below customary standards (0.39). Although two items were slightly below the standard for elementary students (items 9 and 13), other items were acceptable (Table 1).

**Table 4:** Goodness-of-fit indices of CFI for the power to live questionnaire.

**Factor structure**	**Subjects**	**χ^2^**	**CFI**	**TLI**	**RMSEA**	**RMSEA 90% CI**	**SRMR**
34-item version	Elementary school students	1197.55^***^	0.84	0.82	0.06	[0.06, 0.07]	0.07
	Junior high school students	1409.03^***^	0.88	0.87	0.06	[0.06, 0.07]	0.07
	Elementary school students' parent	1365.43^***^	0.82	0.80	0.07	[0.07,0.07]	0.08
16-item version	Elementary school students	122.92**	0.97	0.95	0.04	[0.03, 0.05]	0.04
	Junior high school students	152.51^***^	0.98	0.96	0.05	[0.04, 0.06]	0.03
	Elementary school students' parent	185.21^***^	0.94	0.90	0.06	[0.05, 0.08]	0.05

CFI, comparative fit index; RMSEA, root-mean-square error of approximation; SRMR, standardized root-mean-square residual; TLI, Tucker–Lewis index.

*** p < 0.001.

### Descriptive statistics and reliability in the 16-item version of the power to live questionnaire

Means, standard deviations, and Cronbach's alpha for each factor of the 16-item version of the power to live questionnaire are shown in Table 5. For elementary school students, a certain degree of reliability was found for leadership (0.72); however, other factors showed a generally low reliability (from 0.47 to 0.62). By contrast, junior high school students had a low value of Cronbach's alpha for stubbornness (0.55) and altruism (0.63); however, generally acceptable reliability coefficients were obtained for other factors (from 0.70 to 80).

**Table 5:** Descriptive statistics and Cronbach's alpha of the power to live questionnaire (16-item factor structure).

		**Elementary school students**			**Junior-high school students**	
	***M***	**SD**	**α**	***M***	**SD**	**α**
1. Leadership	4.85	2.49	0.72	5.88	2.69	0.78
2. Problem-solving	5.82	2.24	0.61	6.99	2.07	0.70
3. Altruism	6.76	2.22	0.64	7.36	2.08	0.66
4. Stubbornness	5.35	2.26	0.50	5.20	2.12	0.55
5. Etiquette	7.42	2.00	0.56	8.55	1.80	0.70
6. Emotional regulation	5.52	2.19	0.63	6.80	2.40	0.70
7. Self-transcendence	7.40	1.97	0.53	7.12	2.19	0.73
8. Active wellbeing	5.92	2.33	0.47	6.65	2.36	0.80

### Confirmatory factor analysis and factor loading in the 16-item version of the power to live questionnaire

The lower part of Table 4 shows the results of CFA in the 16-item version factor structure. For both elementary and junior high school students, all items exceeded conventional factor loading criteria (>0.40; Supplementary Table 1). Adequate goodness-of-fit indices were observed in elementary school students (CFI = 0.97, TLI = 0.95, RMSEA = 0.04, and SRMR = 0.04). Correlation coefficients (Spearman's ρ) between the two items measuring each factor were ρ = 0.56, 0.45, 0.48, 0.33, 0.39, 0.46, 0.37, and 0.31, respectively, for factors 1 to 8. The correlation coefficients between the 34- and 16-item versions were *r* = 0.90, 0.83, 0.89, 0.81, 0.92, 0.91, 0.48, and 0.90, respectively, for factors 1 to 8 (>0.80 for all, except factor 7).

Regarding CFA for junior high school students, the trends for goodness-of-fit indices were similar to those for elementary school students (CFI = 0.98, TLI = 0.96, RMSEA = 0.05, and SRMR = 0.03). The correlation coefficients (Spearman's ρ) between the two items measuring each factor were 0.62, 0.52, 0.45, 0.37, 0.48, 0.57, 0.55, and 0.52, respectively, for factors 1 to 8. The correlation coefficients between the 34- and 16-item versions were *r* = 0.93, 0.90, 0.91, 0.86, 0.93, 0.91, 0.90, and 0.95. The results of CFA analysis for the 16-item version of the power to live questionnaire for elementary and junior high school students are shown in Supplementary Table 1.

### Relationship between parental report and child report of the power to live questionnaire

The results of CFA for parental rating of the power to live questionnaire (Table 4) revealed that based on the 34-item version, the goodness-of-fit indices were CFI = 0.82, TLI = 0.80, RMSEA = 0.07, and SRMR = 0.08). For the 16-item version, acceptable levels of fitness of good indices were obtained (CFI = 0.94, TLI = 0.90, RMSEA = 0.06, and SRMR = 0.05). The correlation coefficients for parental and self-ratings of the corresponding factors were all small to moderately positive in the 34-item version (*r* = 0.46, 0.29, 0.47, 0.46, 0.39, 0.30, 0.20, and 0.26, respectively, for factors 1–8; all *p* < 0.001). The 16-item version also showed small to moderate positive correlations (factors 1–8; *r* = 0.48, 0.25, 0.40, 0.41, 0.41, 0.26, 0.18, and 0.16, respectively; all *p* < 0.001).

### Homogeneity of power to live factor structure across schools

Multigroup CFA were conducted combining datasets from elementary school and junior high school students. The results are summarized in Table 6. The results regarding the goodness-of-fit indices and differences in chi square values between models indicate that the configural invariance model was supported for both the 34- and 16-item versions of the structure, suggesting that the factor loading for each power to live factor varied between elementary and junior high schools.

**Table 6:** Results of multigroup CFA for the power to live questionnaire.

**Factor structure**	**Model**	**χ^2^**	**df**	**Δχ^2^**	**df**	***p***	**CFI**	**TLI**	**AIC**	**BIC**	**RMSEA**	**RMSEA 90%CI**	**SRMR**
34-item version	Configural invariance	2606.58	998				0.87	0.85	84769.24	85998.06	0.06	[0.06, 0.07]	0.07
	Weak invariance	2681.13	1024	74.54	26	<0.000	0.86	0.85	84791.78	85897.72	0.06	[0.06, 0.07]	0.07
	Strong invariance	3146.37	1050	465.25	26	<0.000	0.83	0.81	85205.03	86188.08	0.07	[0.07, 0.07]	0.08
16-item version	Configural invariance	275.43	152				0.97	0.96	40186.46	40904.85	0.04	[0.04,0.05]	0.03
	Weak invariance	289.66	160	14.23	8	0.08	0.97	0.96	40184.69	40865.27	0.04	[0.04, 0.05]	0.04
	Strong invariance	350.12	168	60.46	8	<0.000	0.96	0.94	40229.15	40871.92	0.05	[0.05,0.06]	0.04

AIC, Akaike information criterion; BIC, Bayesian information criterion; CI, confidence interval, CFA, confirmatory factor analysis, CFI, comparative fit index, RMSEA, root-mean-square error of approximation, SRMR: standardized root-mean-square residual; TLI, Tucker–Lewis index.

To explore age and gender differences among power to live factors, we conducted multiple regression analyses, where each power to live factor was the objective variable and age, gender, and age-by-gender interaction terms were subjective variables (Supplementary Table 2). Gender was coded 0 for females and 1 for males. The results revealed a statistically significant positive effect of age on leadership, problem-solving, stubbornness, etiquette, emotional regulation, active wellbeing. For altruism, by contrast, the negative partial regression coefficient for gender was significant; however, the effects of age and age-by-gender interaction effects were not significant. None of the explanatory variables were significant for self-transcendence.

### Relationship between awareness of disaster prevention and power to live in elementary school students

Because of the low reliability of each factor of the 16-item version of the power to live questionnaire, we only report the relationships between the 34-item version power to live factors and awareness of disaster prevention. Results for the 16-item version are shown in Supplementary Table 3. Table 2 shows the relationship between elementary school students' awareness of disaster prevention and each power to live factor. Significant positive correlations were found between Q1 and Q2 regarding self-evaluation of one's own or family members' ability to evacuate safely in the event of a disaster and altruism. In addition, we observed a significant negative correlation between Q3, which is regarding fear of disasters, and emotional regulation. Q5, which is on proactiveness, showed a positive relationship with etiquette and problem-solving. Q6 and Q7, which are related to communication with family members about disaster prevention, were positively correlated with self-transcendence. Q8 was positively correlated with etiquette. No significant correlation coefficients were found for leadership, stubbornness, and active well-being.

## Discussions

In this study, we examined the validity of the factor structure and its reliability in elementary and junior high school students regarding the power to live in overcoming sustained challenges, such as natural disasters. In addition, the relationship between awareness of disaster prevention and power to live was explored with the intention of adapting the results to disaster prevention education. The results of CFA for the 34-item and 16-item versions of power to live questionnaires were acceptable for both elementary and junior high school students. In elementary school students, the results were confirmed by parental ratings. These results support the structural validity of the power to live in elementary and junior-high school students. However, in terms of reliability, the results suggest that some factors are not stable, especially for elementary school students. Among junior high school students, many reliability coefficients increased, but stubbornness remained insufficiently reliable. Leadership, problem-solving, altruism, and emotional regulation are suggested to be relatively stable at the elementary school age. When these factors were examined in relation to disaster prevention awareness, correlations were found between these factors and items such as fear of disasters and preparedness in cooperation with family members. Based on these results, this study partially clarified the formation process of the power to live extracted from the survivors of the 2011 Great East Japan earthquake and tsunami. In addition, we were able to identify factors closely related to early disaster prevention awareness.

### Factor structure and reliability of the power to live in elementary and junior high school students

As mentioned, the power to live in elementary and junior high school students was supported in terms of factorial validity; however, some factors were found to be unreliable. In the self-report, stubbornness was consistently unreliable regardless of school type or scale type (34-item or 16-item version). In elementary school students, the 34-item version was not sufficiently reliable for etiquette, self-transcendence, and active well-being. More factors were unstable in the 16-item version, but this is due in large part to the nature of Cronbach's alpha that the number of items is affected (25). The results from multigroup CFA, which suggested the configural invariance model, indicated that some power to live factors, such as stubbornness, etiquette, self-transcendence, and active wellbeing, vary across school types.

The following possibilities exist for the lack of stability in both elementary and junior high school students for stubbornness. First, their beliefs or desires, which are a core part of stubbornness, were in the process of formation. Stubbornness is the tendency to stick to one's beliefs and desires and is assumed to be related to persistence in difficult situations. Elementary and junior high school students may have been in the process of self-formation (26, 27), and thus, had lower reliability coefficients for stubbornness. Second, cognitive immaturity of students may be confounded by low alpha values. This reason might also be involved in problem-solving, which had marginal reliability in elementary school students.

Etiquette and active well-being had lower reliability among elementary school students and relatively higher reliability among junior high school students. These results may be related to differences in age and parental involvement between elementary and junior high school students (28, 29). Elementary school students need adult help to maintain their own health and learn etiquette. An increased risk of physical and mental health problems was reported in children required to care for themselves more from an early age (30). Conversely, learning customary manners and developing habits for one's well-being during elementary and junior high school years may underpin an aspect of the power to live.

In a study on self-transcendence in elementary school students, a lower reliability coefficient was reported than that in adults (31). In self-transcendence, which concerns the perception of the self in the world and society in terms of spiritual significance, a long process of experience-dependent development from early childhood is assumed (10, 32). Knowledge of social conventions and a certain degree of self-establishment are considered to be the primordial stages of self-transcendence (33). Moreover, it has been suggested that the changes of self-transcendence are complex even after adolescence (34). This may be a reason for the low reliability of self-transcendence in elementary school children.

### Relationship between power to live and disaster prevention awareness in elementary school students

We explored how the power to live is related to the awareness of disaster prevention, which will be fostered through disaster prevention education, in elementary school students. We found a relationship between disaster awareness questions and the factors of the power to live questionnaire.

Regarding the fear of disasters, a reasonable correlation was found between lower fear of disaster (Q3) and higher score in emotional regulation. A positive correlation was found between self-assessment of ability to evacuate themselves (Q1) and their families (Q2) during a disaster and altruism. This result is consistent with studies that show that evacuation from disasters involves a variety of social support and that altruism is related to it (6, 35). Awareness of the possibility of disaster (Q5) was related to problem-solving, and etiquette. Learning about possible disasters in the community and how to protect oneself and preparing for disasters with family are the basics of disaster prevention (36). Our results suggested that several factors of power to live were related to awareness for disaster prevention efforts.

Finally, leadership showed a weak positive correlation with items such as awareness of one's own evacuation (Q1) and proactive attitude toward disaster prevention (Q5 and Q6), but it was not statistically significant, despite consistently adequate reliability in all CFA models. The relationship between leadership and resilience was identified as a characteristic that contributes to facilitating the resolution of long-term difficulties (37). Even elementary school students have been observed successfully leading groups (38, 39). Leadership was relatively stable as a factor, but may have been less likely to be associated with awareness of disaster prevention in elementary school students who were influenced by family members or other adults in leadership roles (40).

### Limitation

It is necessary to verify concurrent validity with other psychological scales and cross-validity in a different sample set. In particular, leadership should be validated in relation to indicators such as role in the class (e.g., whether the student is a member of the class council). In addition, the number of participants in this study, which examined the relationship between each power to live factor and awareness toward disaster prevention, was small. Further validation with a larger number of participants is needed.

For methodological reasons, we did not evaluate first- and second-grade elementary school students. Research on how the eight factors extracted from adult data are formed, including studies using different factor structures and methodologies, must clarify the formation process of the power to live. In relation to disaster education, it will be useful to examine the relationship between the power to live in disasters and the acquisition of knowledge through disaster education and compare scores before and after the implementation of disaster education.

In elementary school students, stubbornness, etiquette, self-transcendence, and active well-being were qualitatively different from those in adults and may have led to low reliability. Future studies must clarify the neurocognitive bases of factors that are formed empirically among power to live factors as well as the environmental factors involved in their formation. Studies are being conducted to examine the cognitive basis of self-transcendence (9, 10). By contrast, leadership, problem-solving, altruism, and emotional regulation were found to be reliable, even among elementary school students. Factors such as executive function (41, 42) and altruism (43), which are believed to be stable from relatively early developmental stages, may related to these factors.

## Conclusion

We examined the psychological traits, termed power to live, that enabled survivors to overcome the challenges in the immediate aftermath of the 2011 Great East Japan earthquake and tsunami and rebuild their lives in elementary and junior high school students. The results of CFA and reliability coefficients indicated that leadership, problem-solving, altruism, and emotional regulation were stable and related to disaster awareness even in the middle and upper grades of elementary school. In junior high school students, all factors (excluding stubbornness), such as etiquette and active wellbeing, were stable. Factors related to cognitive skills, such as problem-solving and altruism, that facilitate building relationships with others, including adults, and emotional regulation, which helps tolerate disaster anxiety and fear, were consistent with factors identified in other studies on disaster prevention. Therefore, the power to live questionnaire has the potential to contribute to disaster education for children and foster resilience. Future research should focus on improving the power to live questionnaire to test the relationship between the questionnaire and disaster prevention education or actual behavior in emergency. In addition, as the subjects were exclusively Japanese, it is necessary to examine whether the structure of the power to live is replicated in other cultures in order to make this questionnaire a useful tool. It would be also useful to examine the aspects of each factor of the power to live that can be cultivated as well as educational programs for this purpose. These considerations could make this scale a useful tool in clarifying the neurocognitive basis and experiential factors involved in long-term resilience, such as disaster recovery.

## Data availability statement

The raw data supporting the conclusions of this article will be made available by the authors, without undue reservation.

## Ethics statement

The studies involving human participants were reviewed and approved by the Institutional Review Board of the International Research Institute of Disaster Science, Tohoku University, Japan. Written informed consent from the participants' legal guardian/next of kin was not required to participate in this study in accordance with the national legislation and the institutional requirements.

## Author contributions

Conceptualization, methodology, writing, review, and editing: MS, TA, AH, AT-I, MY, RI, and YM. Formal analysis and writing original draft preparation: YM. All authors contributed to the article and approved the submitted version.
