# Supplementary material for: Does the eight-factor “power to live” in disaster exist since childhood?
Source: Front Public Health. 2022 Dec 12;10:1022939. doi: 10.3389/fpubh.2022.1022939 (PMC9791042; doi:10.3389/fpubh.2022.1022939)
Supplement: Supplementary file 1 [file Table_1.docx]

Supplementary Table 1. Power to live questionnaire and factor loading in elementary and junior high school students (16-itemt version factor structure).

| Item | Elemen-tary school student | Junior high school student |
| --- | --- | --- |
| Factor 1. Leadership | | |
| To resolve problems, I gather together everyone involve to discuss the matter. | 0.75 | 0.78 |
| In everyday life, I often take the initiative to gather people together. | 0.75 | 0.81 |
| Factor 2. Problem-solving | | |
| When I am fretting about what I should do, I compare several alternative actions. | 0.63 | 0.64 |
| To resolve a problem, I first of all initiate action. | 0.70 | 0.83 |
| Factor 3. Altruism | | |
| I like it when other people rely on me and are grateful to me. | 0.66 | 0.62 |
| When I see someone having trouble, I have to help them. | 0.73 | 0.79 |
| Factor 4. Stubbornness | | |
| I am stubborn and always get my own way. | 0.46 | 0.66 |
| I unhesitatingly say whatever it is I want to say. | 0.74 | 0.57 |
| Factor 5. Etiquette | | |
| On a daily basis, I take the initiative in greeting family members and people living in the neighborhood. | 0.65 | 0.69 |
| When someone has helped me or been kind to me, I clearly convey my feelings of gratitude. | 0.60 | 0.78 |
| Factor 6. Emotional regulation | | |
| During difficult times, I endeavor not to brood. | 0.60 | 0.67 |
| During difficult times, I endeavor to think positively, telling myself that this experience will benefit me in the future. | 0.77 | 0.81 |
| Factor 7. Self-transcendence | | |
| I am aware that I am alive, and have a sense of responsibility in living. | 0.58 | 0.69 |
| I am aware of the path and teachings I should follow as a person. | 0.62 | 0.84 |
| Factor 8. Active well-being | | |
| In everyday life, I have habitual practices that are essential for relieving stress of giving me a change of pace. | 0.49 | 0.62 |
| In everyday life, I endeavor to find opportunities to acquire new knowledge, skills, and attitudes. | 0.64 | 0.85 |

Supplementary Table 2. Multiple regression analyses of the effect of age and sex on the power to live.

|  | Age | | | Gender | | | Age-by-Gender interaction | | | R^2^ | Adjusted R^2^ |
| --- | --- | --- | --- | --- | --- | --- | --- | --- | --- | --- | --- |
|  | *β* | *t* | *p* | *β* | *t* | *p* | *β* | *t* | *p* |  |  |
| 1. Leadership | 0.11 | 2.16 | 0.03 | 0.00 | 0.05 | 0.96 | 0.05 | 0.78 | 0.44 | 0.02 | 0.02 |
| 2. Problem-solving | 0.32 | 6.88 | <0.000 | 0.01 | 0.23 | 0.82 | 0.03 | 0.49 | 0.63 | 0.12 | 0.11 |
| 3. Altruism | 0.05 | 1.06 | 0.29 | −0.35 | −5.16 | <0.000 | 0.07 | 1.03 | 0.30 | 0.04 | 0.04 |
| 4. Stubbornness | 0.12 | 2.47 | 0.01 | 0.04 | 0.59 | 0.56 | −0.05 | −0.68 | 0.5 | 0.01 | 0.01 |
| 5. Etiquette | 0.24 | 4.96 | <0.000 | 0.05 | 0.73 | 0.47 | 0.01 | 0.13 | 0.9 | 0.06 | 0.06 |
| 6. Emotional regulation | 0.28 | 5.83 | <0.000 | 0.09 | 1.31 | 0.19 | 0.06 | 0.96 | 0.34 | 0.10 | 0.10 |
| 7. Self-transcendence | −0.01 | −0.15 | 0.88 | −0.06 | −0.88 | 0.38 | 0.02 | 0.33 | 0.74 | 0 | 0 |
| 8. Active well-being | 0.19 | 3.79 | <0.000 | 0.03 | 0.48 | 0.63 | 0.03 | 0.46 | 0.64 | 0.04 | 0.04 |

Note: β was standardized partial regression coefficient

Supplementary Table 3. Correlation coefficients between disaster prevention awareness and power to live factors (16-item version).

| Item | Leader-  Ship | Problem-solving | Altruism | Stubborn-ness | Eti-quette | Emotio-nal regulation | Self-  Transcen-dence | Active  well-being |
| --- | --- | --- | --- | --- | --- | --- | --- | --- |
| Q1. Do you think you could evacuate safely if an earthquake, tsunami, typhoons, heavy rain, or volcanic eruption were to occur? | 0.23 | 0.12 | 0.34^†^ | −0.30 | 0.16 | 0.20 | 0.45^*^ | −0.10 |
| Q2. Do you think your family could evacuate safely if an earthquake, tsunami, typhoons, heavy rain, or volcanic eruption were to occur? | 0.00 | 0.12 | 0.45^*^ | −0.10 | 0.02 | −0.07 | 0.22 | −0.04 |
| Q3. Are you scared of natural disasters, like earthquakes, tsunamis, typhoons, heavy rain, and volcanic eruptions? | −0.27 | −0.27 | −0.17 | 0.13 | −0.09 | −0.43^*^ | −0.16 | −0.19 |
| Q4. Do you think you may get injured if a natural disaster were to occur? | −0.12 | 0.14 | 0.16 | −0.11 | 0.22 | −0.11 | 0.11 | 0.08 |
| Q5. Do you think natural disasters will occur in your area? | 0.25 | 0.33 | 0.22 | −0.45^*^ | 0.40^†^ | 0.27 | 0.19 | 0.23 |
| Q6. Do you think you need to talk with your family to decide what to do in the event of a natural disaster? | 0.05 | −0.09 | 0.13 | 0.01 | 0.14 | −0.03 | 0.40^†^ | 0.06 |
| Q7. Do you think your family would become safer if you talked and made promises to improve preparedness? | 0.01 | 0.30 | 0.22 | −0.39^†^ | 0.51^*^ | 0.00 | 0.44^*^ | 0.07 |

^†^*p* < 0.10 **p* < 0.05
